# Antiviral Action of Hydromethanolic Extract of Geopropolis from *Scaptotrigona postica* against Antiherpes Simplex Virus (HSV-1)

**DOI:** 10.1155/2015/296086

**Published:** 2015-03-11

**Authors:** Guilherme Rabelo Coelho, Ronaldo Zucatelli Mendonça, Karina de Senna Vilar, Cristina Adelaide Figueiredo, Juliana Cuoco Badari, Noemi Taniwaki, Gisleine Namiyama, Maria Isabel de Oliveira, Suely Pires Curti, Patricia Evelyn Silva, Giuseppina Negri

**Affiliations:** ^1^Instituto Butantan, Avenida Vital Brasil, No. 1500, Butantã, 05503-900 São Paulo, SP, Brazil; ^2^Instituto Adolfo Lutz, Avenida Dr. Arnaldo 355, 01246-900 São Paulo, SP, Brazil; ^3^CEBRID, Departamento de Medicina Preventiva, Universidade Federal de São Paulo, Rua Botucatu 740, Vila Clementino, 04023-900 São Paulo, SP, Brazil

## Abstract

The studies on chemical composition and biological activity of propolis had focused mainly on species *Apis mellifera* L. (Hymenoptera: Apidae). There are few studies about the uncommon propolis collected by stingless bees of the Meliponini tribe known as geopropolis. The geopropolis from *Scaptotrigona postica* was collected in the region of Barra do Corda, Maranhão state, Brazil. The chemical analysis of hydromethanolic extract of this geopropolis (HMG) was carried out through HPLC-DAD-ESI-MS/MS and the main constituents found were pyrrolizidine alkaloids and C-glycosyl flavones. The presence of alkaloids in extracts of propolis is detected for the first time in this sample. The antiviral activity of HMG was evaluated through viral DNA quantification experiments and electron microscopy experiments. Quantification of viral DNA from herpes virus showed reduction of about 98% in all conditions and concentration tested of the HMG extract. The results obtained were corroborated by transmission electron microscopy, in which the images did not show particle or viral replication complex. The antiviral activity of C-glycosyl flavones was reported for a variety of viruses, being observed at different points in the viral replication. This work is the first report about the antiviral activity of geopropolis from *Scaptotrigona postica, in vitro*, against antiherpes simplex virus (HSV).

## 1. Introduction

Propolis is a resinous material comprising plant exudates and wax used by bees for sealing the hive and as protection against microorganisms [1, 2]. The studies about chemical composition and biological activity of propolis had focused mainly on species* Apis mellifera* L. (Hymenoptera: Apidae). The uncommon propolis collected by stingless bees of the Meliponini tribe is a mixture of resin, wax, and soil known as geopropolis. Stingless bees are widely found in tropical and subtropical areas worldwide [3, 4].

The geopropolis from* Scaptotrigona postica* had been used popularly in the region of Barra do Corda, Maranhão state, Brazil, in the form of ointment in the treatment of tumors and wound healing [5, 6] but there is no information on its chemical composition and biological activity. The chemical composition of the geopropolis of some countries, including Brazil, was analyzed recently. Eleven compounds belonging to the classes of phenolic acids and hydrolyzable tannins (gallotannins and ellagitannins) [4] and benzophenones [7] were found from geopropolis of* Melipona scutellaris*. Flavonoids glycosides were found in geopropolis from two species of stingless Amazonian bees,* Melipona interrupta* and* Melipona seminigra* [8]. Phenylpropanoids and flavonoids were found in geopropolis from* Melipona subnitida* (jandaira) stingless bee [9] and aromatic acids; phenolic compounds and terpenes are detected from geopropolis of the stingless bee* Melipona orbignyi* (Hymenoptera, Apidae) found in Mato Grosso do Sul, Brazil [10].

Flavones-di-C-glycosides, caffeoylquinic acid derivatives, and polyprenylated benzophenones had been reported in propolis [11–13]. The similarity in chemical composition of propolis and geopropolis was attributed to the fact that the two bees (Africanized and stingless bees) produce this bee product using resin collected from plants. The C-methylated flavanones that were detected in geopropolis from Australian stingless bees (*Tetragonula carbonaria*, Meliponini) were also found in extracts from* Corymbia torelliana* (Myrtaceae) fruit resins, of which probably* T. carbonaria* collected the resin for the production of its geopropolis [14].

Pyrrolizidine alkaloids, a diverse class of monoesters, are generally found in plants from the families Asteraceae, Boraginaceae, and Fabaceae and are present approximately in 6000 flowering plants species worldwide [15]. The presence of 1,2-dihydropyrrolizidine alkaloids had been observed in bee products, such as honeys and pollen. Echimidine is one of the main alkaloids reported in honey [15–18]. There are no reports about the presence of alkaloids in propolis [2].

Herpes simplex viruses (HSV) are part of the alphaherpesvirus subfamily of herpes viruses. The incidence of diseases caused by herpes simplex virus (HSV) types 1 and 2 has increased in recent years [19]. HSV-1 and HSV-2 are closely related to ancient human pathogens responsible for a number of diseases, including oral and genital ulcerations, virally induced blindness, viral encephalitis, and disseminated infections of neonates [19, 20]. HSV-1 suppresses the interferon (IFN) signaling pathway of infection at multiple sites in order to evade host defense mechanisms [19]. There are three ways to control HSV infections using anti-HSV drugs, microbicides, and vaccine. Nowadays, the standard therapy for the management of HSV infections includes acyclovir and penciclovir with their respective prodrugs valacyclovir and famciclovir [19, 20]. The development of the novel strategies to control HSV is a global public health priority.

The aim of this work was to evaluate the chemical composition and antiviral activity of the hydromethanolic extract of geopropolis (HMG) from* Scaptotrigona postica* against antiherpes simplex virus (HSV).

## 2. Material and Methods

### 2.1. Cells

The Vero cells (African green monkey kidney—ATCC CCL-81) were grown in 75 cm^2^ plastic cell culture flasks in DMEM medium (Dulbecco's Minimum Eagle Essential Medium) supplemented with 10% inactive fetal bovine serum (FBS) and 20 mM L-glutamine (Invitrogen, USA).

### 2.2. Determination of the Virus Infectious Dose

The confluent monolayers were dispersed with 0.2% trypsin and 0.02% versene and added in DMEM growth medium with 100 IU/mL penicillin G and 100 mg/mL streptomycin. For the preparation of 96-well plates, the cell suspension was diluted to 2.0 × 10^4^ cells/mL. Plates were seeded with 200 *μ*L of suspension and incubated at 37°C in a humidified 5% CO_2_ atmosphere. Herpes simplex virus strain (McIntyre) stock virus was quantified by medium tissue—culture infections with 0.01 moi. (multiplicity of infection) on cell cultures. The confluent cell cultures were inoculated with 100 *μ*L diluted virus in quadruplicates. After 1 hour adsorption at room temperature, each well received 200 *μ*L of DMEM medium with 2% FBS. Uninfected cultures were also prepared and treated identically as controls. Plate cultures were observed for CPE daily during seven days when the test was concluded. Fifty percent infectivity end points were calculated by the method of Reed and Muench [21]. All titers were given as log 10 TCID_50_ per 0.1 mL of virus.

### 2.3. MTT Assay

The cell viability assay was based on MTT reduction in the mitochondria by enzymatic action which generated a colored product. Vero cells were cultured in 96-well plates in DMEM supplemented with 5% of fetal bovine serum. After 48 h, HMG with concentrations ranging from 10 mg/mL to 0.1 mg/mL was added to the wells culture medium. After 24 hours of exposure the supernatant was discarded and MTT at concentration of 500 microgram/mL was added to the growth medium for 4 hours. After this, the culture medium was removed and 100 mL of DMSO was added. After addition of dimethyl sulfoxide (DMSO), the plates were shaken for 30 minutes and then were read on a spectrophotometer at 570 nm.

### 2.4. Antiviral Effect of Geopropolis on Infected Cells

Vero cells were grown to approximately 90% confluence in 96-well plates in DMEM supplemented with 2 mM L-glutamine and 10% of FBS. Plates were incubated at 37°C in a humidified 5% CO_2_ atmosphere. The confluent cells were infected with HSV-1 at a concentration of 10^−8^ and monitored for cytopathic effects during 3 days. HMG was added to the cells at 3 h prior to virus infections, 1 h after virus infection, and virucida. These antiviral screenings were repeated independently three times with three concentrations of HMG (100, 10, and 1 *μ*g/mL). After this, the determination of the geopropolis effect on the infected cells was carried out using Real-Time PCR.

### 2.5. Quantitative Real-Time PCR Assay

Genomic DNAs including viral DNAs were isolated from the harvested cells using the MagNA Pure extractor (Roche, Basel, Switzerland) according to the manufacturer's instructions. The forward primer sequence for HSV-1 is 5′-TGGGACACATGCCTTCTTGG while the reverse sequence is 5′-ACCCTTAGTCAGACTCTGTTACTTACCC with amplicon size of 147 bp [22]. For the Real-Time PCR, the 20 *μ*L reaction mixture contained 12 *μ*L of the SYBR Green PCR Master Mix (Applied Biosystems, Foster City, CA, USA), 10*μ*M of each primer, 6.5 *μ*L H_2_O, and 5 *μ*L of cDNA. The assay was performed using the ABI 7300 Real-Time PCR Systems (Applied Biosystems, Foster City, USA) under the following conditions: 94°C for 5 min, followed by 35 cycles of 94°C for 30 s, 57°C for 20 s, and 72°C for 40 s. Standard curves were prepared by qPCR using serial dilution of known copies number of purified amplification product for HSV-1. The copy number of the samples was calculated from the standard curves [23]. Percentage of reduction was defined as [copy number of infected − copy number of treated]/copy number of infected × 100. All experiments were made in triplicate.

### 2.6. Transmission Electron Microscopy (TEM)

The Vero cells were cultivated on Aclar film [24] and after two days cells were infected with the sample from virucida (virus incubated with propolis during 1 h) and with HSV at concentration of 10^−8^. After 48 h, the cultures were fixed* in situ* using 2.5% glutaraldehyde (Sigma, USA) in 0.1 M sodium cacodylate buffer and pH 7.2 for 1 h at 4°C. After they were rinsed twice with cacodylate buffer, the cultures were post-fixed in a solution containing 1% osmium tetroxide, 0.8% potassium ferrocyanide, and 5 mM calcium chloride, washed in 0.1 M sodium cacodylate buffer, dehydrated in graded acetone, and embedded in epoxy resin. Ultrathin sections were stained using uranyl acetate and lead citrate and examined through a transmission electron microscope JEM-1011 (JEOL, Japan).

### 2.7. Direct Electron Microscopy (DEM)

The supernatant cells infected with herpes virus at concentration of 10^−8^ and treated with virucida were resuspended in 50 mL of phosphate-buffered saline (PBS) at pH 7.2. One drop of the suspension was put on EM grid and submitted to negative staining technique with 2% potassium phosphotungstate (PTK) at pH 6.4. The grids were examined and the viruses were documented in a JEM-1011 (JEOL, Japan) electron microscope [22].

### 2.8. Separation and Identification of the Constituents of the Hydromethanolic Extract of Geopropolis (HMG) from* Scaptotrigona postica*

#### 2.8.1. Geopropolis Sample from* Scaptotrigona postica*

The geopropolis sample from* Scaptotrigona postica* was collected in the region of Barra do Corda, Maranhão state, Brazil. The ethanolic extract of geopropolis was provided by beekeeper. The geopropolis (1 Kg) was extracted by maceration in 1 L of cereal alcohol during 3 months and after this the crude preparation was filtered first through cotton and then through filter. The alcoholic extract was concentrated and stored at freezer until sample workup. For biological tests, the ethanolic extract prepared by beekeeper was dissolved in hexane, ethyl acetate, and a mixture of water/methanol (1 : 2) that obtained a fraction rich of hydrossoluble compounds, which was dried and, after this, dissolved in water Milli-Q.

#### 2.8.2. Analysis of the Hydromethanolic Extract from Geopropolis (HMG) Using Fourier Transform Infrared Spectrometry (FTIR)

FTIR spectrum was recorded at room temperature (ca. 25°C) using a Bomem spectrometer by scanning over the frequency range 4000–400 cm^−1^ at a resolution of 5 cm^−1^ using pastilles of KBr.

#### 2.8.3. Reversed Phase HPLC-DAD-ESI-MS/MS Analysis

The HPLC-DAD-ESI-MS/MS analysis was conducted on DADSPD-M10AVP Shimadzu system equipped with a photodiode array detector coupled to Esquire 3000 Plus (Bruker Daltonics), which consisted of two LC-20AD pumps, SPD-20A diode array detector, CTO-20A column oven, and SIL 20AC autoinjector (Shimadzu Corporation Kyoto, Japan). The mass detector was a quadrupole ion trap equipped with atmospheric pressure ionization source through electrospray ionization interface, which was operated in the full scan MS/MS mode. All the operations, acquisition, and data analysis were controlled by CBM-20A software. The HMG was dissolved in methanol : water (80 : 20) v/v and filtered with 0.45 *μ*m filter, before 31.2 *μ*L was injected into the HPLC system. The peaks were monitored with diode array detection at wavelengths of 254 and 300 nm. The mobile phases consisted of two solvents: eluent A (0.1% aq. formic acid in Milli-Q water) and eluent B (acetonitrile). Constituents were separated using a reverse phase, Phenomenex Gemini C-18 (250 × 4.6 mm, 5 um), connected to a guard column. The elution started with 10% B in A; at 10 min it reached 20% B in A; at 20 min, 40% B in A; at 30 min, 60% B in A; at 40 min, 80% B in A; at 50 min, 100% B in A; and finally it returned to the initial conditions (10% B) to reequilibrate the column prior to another run. The flow rate was kept constant at 1.0 mL min^−1^ and the temperature of the column was maintained at 40°C. The ionization conditions were adjusted as follows: electrospray ionization was performed using an ion source voltage of 40 V and a capillary offset voltage of 4000 V. The full scan mass acquisition was performed using electrospray ionization in the positive ionization mode by scanning from *m*/*z* 100–1200 u. Helium was used as the collision gas and nitrogen as the nebulizing gas. Nebulization was aided with a coaxial nitrogen sheath gas provided at a pressure of 27 psi. Desolvation was assisted using a counter current nitrogen flow set at a flux of 7.0 L/min and a capillary temperature of 320°C. The data dependent MS/MS events were performed on the most intense ions detected in full scan MS. Maximum accumulation time of ion trap and the numbers of MS to obtain the MS average spectra were set at 30 and 3 MS, respectively. All the compounds were characterized by the interpretation of their UV spectra and mass spectra data obtained through ESI-MS and ESI-MS/MS and also taking into account the MS data provided by the literature.

## 3. Results

### 3.1. Determination of the Virus Infectious Dose

The exposure of cell cultures to concentrations up to 2.5 mg/mL of the HMG did not show significant reduction in cell viability.

### 3.2. Quantitative Real-Time PCR Assay

**Figure 1:**
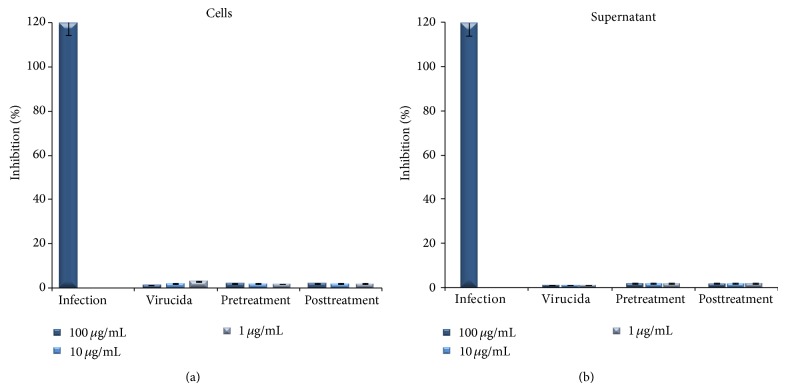
Herpes transcription level cells treated with propolis and measured by Real-Time PCR. Total DNA was performed at 3 hours before infection (pretreatment), 1 hour postinfection (posttreatment), and virucida. (a) Cells lysate. (b) Supernatant. Antiviral activity percentage and relative DNA viral quantification.

**Figure 2:**
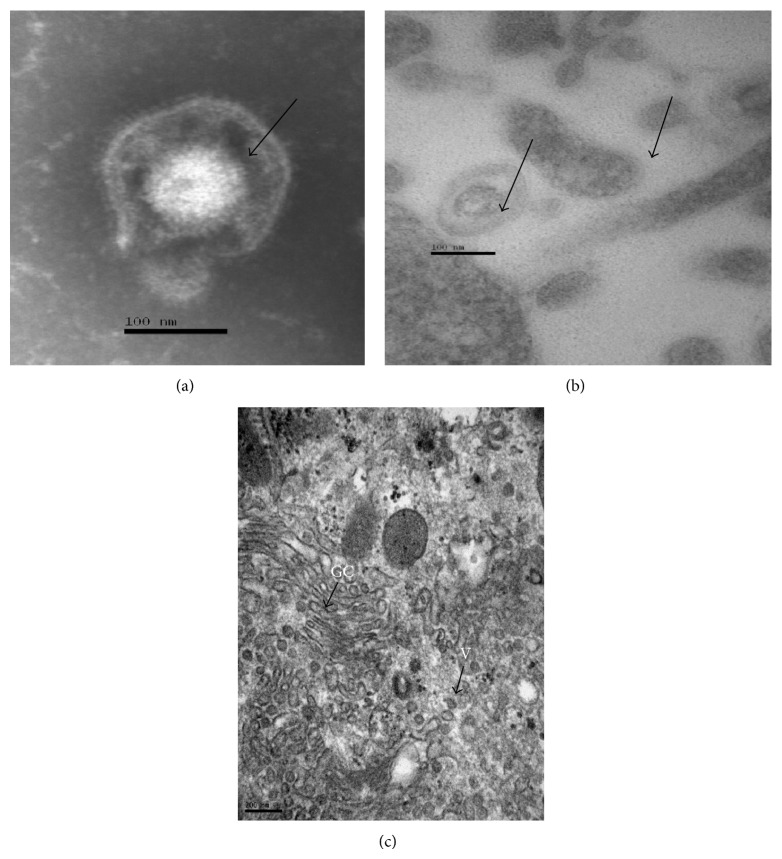
Vero cells were cultivated on Aclar film and were inoculated with HSV-1 or virucida sample after 48 hrs. (a) Vero cells were inoculated with HSV-1 and processed by DEM. Note particle viral. (b) Vero cells were inoculated with HSV-1 and processed by TEM. Note the presence of a typical particle viral. (c) Vero cells were inoculated with virucida sample and processed by TEM. Note Complex the Golgi, vesicles. Not found the HSV like particles.

The antiviral activity of geopropolis against HSV-1 was confirmed by qPCR Real-Time. The HMG was added into each designed well at 3 h before infection (pretreatment), 1 h after infection (posttreatment), and virucida at the concentrations (100, 10, and 1 *μ*g/mL). The results showed that HMG significantly reduced the number of copies of HSV-1 genomic DNA in the supernatant and in the lysate cell (Figures 1(a) and 1(b)). All concentrations tested against HSV-1 through pre-, post-, and virucida treatment were found to be most effective in inhibiting HSV-1 viral replication. This suggested that the geopropolis inhibited the events in the early infection, such as viral binding and viral entry into cells as well as the viral replication. Beside this, in the cells inoculated with virucida sample and processed by DEM and TEM, was not detected the HSV like particles into the cytoplasm from Vero cells analyzed, as can be seen in Figure 2.

### 3.3. Chemical Characterization of Constituents from Geopropolis

#### 3.3.1. Analysis of Hydromethanolic Extract from Geopropolis (HMG) Using Fourier Transform Infrared Spectrometry (FTIR)

Fourier Transform Infrared Spectroscopy (FTIR) is a fast, accurate, and nondestructive technique used as a powerful analytical tool in many fields. The absorption bands observed in the FTIR spectrum [*υ*Max cm^−1^ (KBr)] for HMG were 3365, 2919, 2362, 2343, 1609, 1414, 1077, 1048, 864, and 675 cm^−1^. The O–H stretching vibrations are sensitive to hydrogen bonding and the existence of an intermolecular hydrogen bond can lower the O–H stretching wavenumber to 3500–3200 cm^−1^. The broad peak at 3365 cm^−1^ was attributed to hydroxyl stretching vibration of flavones [25, 26].

The peak at 1609 cm^−1^ was attributed to a carbonyl stretch of flavones, that occur at lower vibrational energies, as a result of decreased bond strengths of the carbonyl bond, due to resonance structures with a C=C bond adjacent to a carbonyl group, resulting in the electron delocalization in the carbonyl and the double bond [25], and at 1048 cm^−1^ was attributed to the vibration of C–OH. The band at 1077 cm^−1^ is a vibration attributed to the C–O–C asymmetric stretching and at 1414 cm^−1^ it was assigned to C=C stretch of the flavones phenyl rings [25, 26]. In apigenin derivatives, the C–C ring stretching vibrations give rise to characteristic bands in IR spectrum covering the spectral range from 1610 to 1300 cm^−1^ [26]. The presence of nitrogen compounds was attributed to absorption bands at 2362 and 2343 cm^−1^.

#### 3.3.2. Analyses of Chemical Constituents through HPLC-DAD-ESI-MS/MS

Mass spectrometry data were acquired through electrospray ionization positive mode.  Table 1 summarizes the following information on peaks observed during RPHPLC-DAD-ESI-MS/MS analyses: retention times (Rt), MS data for protonated molecules, and proposed structures. Constituents, mainly pyrrolizidine alkaloids, were tentatively identified by the similarity of their fragmentation pattern with known compounds reported in the literature data.

The sugar residues of flavonoids glycosides are mainly hexoses (glucose and galactose), 6-deoxyhexoses (rhamnose and furanose), pentoses (arabinose and xylose), and uronic acids (glucuronic acid and galacturonic acid). Glucose, rhamnose, xylose, and arabinose are the more common sugar [27, 28]. Flavonoid* O*-glycosides are bounded to sugar with formation of an acid labile glycosidic O–C bond and their fragmentations patterns involve the heterocyclic cleavage at the glycosidic* O*-linkage (hemiacetal O–C bond) with a concomitant H-rearrangement, leading to the elimination of the saccharide residue [27, 28].

Glycosylation also occurs by direct linkage of the sugar to the basic nucleus of the flavonoid, which is stable towards acid hydrolysis. The main fragmentations take place in the sugar, which possess the weakest bonds, that observed the cross-ring cleavages of the saccharide residue through the successive loss of molecules of water [29–32]. Two sugars can be attached to the flavonoid aglycone, either at two different positions (di-O-glycosides, di-C-glycosides, and di-C,O-glycosides) or at the same position (O-diglycosides, C,O-diglycosides) [27–32]. Sodium adduct ions [M + Na]^+^ at 22 u are often detected in first-order mass spectra obtained with ESI in the positive ion mode [27].

The main flavonoids found in HMG were flavones di-C-glycosides (Table 1). The ESI-MS/MS spectra of protonated molecules of flavones di-*C*-glycosides** 6–11**,** 13**,** 14**, and** 17** displayed neutral losses of water (18 u) as fragments [27–32]. The main flavone di-C-glycoside, compound** 14**, exhibited protonated molecule at *m*/*z* 595 and sodiated molecule [M + Na]^+^ at *m*/*z* 617, respectively. Its ESI-MS/MS spectrum in positive ion mode produced fragment ions at 577, 559, 541, 529, 511, 499, 475, and 457 obtained through the loss of water (Table 1). Compound** 17** exhibited protonated molecule at *m*/*z* 565, which after MS/MS experiments exhibited fragment ions at *m*/*z* 547, 529, 511, and 427. According to literature data [29–32] compound** 14** was characterized as apigenin-6,8-di-*C*-glucoside, also known as vicenin-2, and compound** 17** was characterized as apigenin-6,8-di-C-arabinoside glucoside, respectively.

Apigenin-di-C-malonyl trihexoside isomers (compounds** 7–9**,** 11**, and** 13**) showed different retention times but the same sodiated molecule at *m*/*z* 865 and protonated molecule at *m*/*z* 843 (Table 1), respectively. The malonyl moiety corresponds to 86 u. Besides this, the ESI-MS/MS spectra of protonated molecule showed the same fragmentation pattern at *m*/*z* 825, 807, 789, 771, 723, 705, and 687 attributed to the loss of water from glucose and/or galactose as saccharides [29–32], which possess different intensities for each compound, as can be seen in Table 1, what could be used to discriminate between these apigenin-di-C-malonyl trihexoside isomers.

Other flavones-di-C-glycosides are acacetin and luteolin derivatives. Compound** 6** showed protonated molecule at *m*/*z* 619, which after MS/MS experiments produced fragment ions at *m*/*z* 601, 583, 565, and 535. Compound** 10** exhibited protonated molecule at *m*/*z* 857, which after MS/MS experiments produced fragment ions at *m*/*z* 839, 803, 737, and 719. Compound** 20** showed protonated molecule at *m*/*z* 595, which after MS/MS experiments produced fragment ions at *m*/*z* 577, 567, and 551. Compounds** 6**,** 10**, and** 20** were tentatively characterized as acacetin-di-C-acetyl dirhamnoside, acacetin-di-C-malonyl trihexoside, and luteolin-8-C-caffeoyl rhamnoside, respectively.

Flavones di-C,O-glycosides were also found in low content. The ESI-MS spectrum of compound** 12** exhibited protonated molecule at *m*/*z* 563, which after MS/MS experiments produced high fragment ion at *m*/*z* 417 [M – H − 146]^−^ which was attributed to the loss of the O-deoxyhexosyl radical (rhamnose moiety) probably linked to phenolic hydroxyl group in 7-position of aglycone [27–32]. In the same way, compound** 18** exhibited protonated molecule at *m*/*z* 547, which after MS/MS experiments produced high fragment ion at *m*/*z* 401 [M – H − 146]^−^. Compounds** 12** and** 18** presented O-glycosylation on the phenolic hydroxyl group of aglycone, which was corroborated by literature data [29–32], and were suggested as acacetin-8-C-arabinoside-7-O-rhamnoside and chrysin-8-C-rhamnoside-7-O-rhamnoside, respectively.

The* trans*-cinnamic acids are esterified at one or more of the hydroxyls at positions 1, 3, 4, and 5 of quinic acid, originating a series of positional isomers [33–35]. For compound** 19**, the ESI-MS spectrum showed protonated molecule at *m*/*z* 517, which after MS/MS experiments gave fragment ions at *m*/*z* 355 (caffeoylquinic acid moiety) and *m*/*z* 337, suggesting di-*O*-caffeoylquinic acid positional isomers. The ESI-MS spectrum of compound** 21** exhibited protonated molecule at *m*/*z* 487. According to literature data [28, 33–35], compound** 19** was characterized as 3,5-di-*O*-caffeoylquinic acid and compound** 21** as caffeoylquinic acid-O-arabinoside, respectively.

Compound** 1** was characterized as catechin-3-O-gallate, based on ESI-MS data, that showed protonated molecule at *m*/*z* 443, which after MS/MS experiments produced fragment ion at *m*/*z* 291 (protonated catechin) attributed to the loss of galloyl moiety (152 u) [36, 37]. Other catechin derivatives, compounds** 15** and** 16**, exhibited sodiated molecules at *m*/*z* 445 and *m*/*z* 459 and protonated molecules at *m*/*z* 423 and *m*/*z* 437, respectively. After MS/MS experiments, both compounds exhibited fragment ions generated by the loss of water molecules, indicating the presence of the sugar molecules linked to the catechin. The main fragmentations take place in the sugar which possess the weakest bonds and the cross-ring cleavages of the saccharide residue are observed through the successive loss of molecules of water as shown in Table 1. According to literature data [36, 37] compounds** 15** and** 16** are tentatively characterized as catechin arabinoside and catechin rhamnoside.

The presence of 1,2-dihydropyrrolizidine alkaloids had been observed in bee products and echimidine is the main alkaloid reported in honey [15–18, 38]. According to Boppré et al. [15–17] these compounds are transferred to the honey by bees that visit the flowers to forage nectar and pollen. However, there are no reports about human poisoning from honey by 1,2-dihydropyrrolizidine alkaloids [15–18, 38]. Pyrrolizidine alkaloids similar to echimidine are detected for the first time in this geopropolis from* Scaptotrigona postica*.

The ESI-MS spectrum of compound** 4** showed protonated molecule at *m*/*z* 430, which after fragmentation produced fragment ions at *m*/*z* 412 attributed to the loss of water (18 u) and at *m*/*z* 385 attributed to the loss of (CH_3_CHOH) group of echimidinylretronecine moiety linked at C-9 of open chain diester type of pyrrolizidine alkaloid. The fragment ion [M + H − 115]^+^ at *m*/*z* 315 was attributed to the loss of methoxy dihydro angelic acid moiety that is present on the C-7 of open chain diester type of pyrrolizidine alkaloid. The loss of angelic acid moiety from echimidine was observed by Boppré et al. [15–17]. For compound** 5**, the ESI-MS spectrum exhibited protonated molecule at *m*/*z* 444, while its ESI-MS/MS spectrum exhibited the same fragmentation pattern as** 4** with fragment ions at *m*/*z* 426 and *m*/*z* 399 attributed to the loss of water (18 u) and the loss of (CH_3_CHOH) group of echimidinylretronecine moiety linked at C-9 of open chain diester type of pyrrolizidine alkaloid. According to literature MS data [15–18, 38] the echimidine derivatives** 4** and** 5** were tentatively identified as 7(3-methoxy-2-methylbutyryl)-9-echimidinylretronecine and 7(3-ethoxy-2-methylbutyryl)-9-echimidinylretronecine, respectively.

## 4. Discussion

Herpes simplex virus type 1 (HSV-1) is an important pathogen for humans, and the discovery of novel effective antiherpetic drugs without adverse effects is of great interest [39]. Acyclovir is the first-line treatment for the management of herpes simplex virus 1 (HSV-1) and virus 2 (HSV-2) diseases. However, long-term administration of this drug for the treatment of chronic infections in the immunocompromised host can lead to the development of acyclovir resistance [19, 20, 39, 40].

For geopropolis from* Scaptotrigona postica* [5, 6], there are no studies about biological activities. In order to examine the antiviral activity of HMG against antiherpes simplex virus, the cells were treated with HMG 1 hour before infection; 3 hours after infection, and virucidal condition, and it was observed that in all conditions and concentrations tested the HMG showed great potential in inhibition of replication of HSV-1. Virucida test results suggest that HMG interfered with virion envelope structures or are masking viral compounds which are necessary for adsorption or entry into host cells. Apparently, free herpes virus is very sensitive to HMG and the inhibition of HSV-1 appears to occur before entering into the cell. In our study, in all conditions and concentrations tested, EMG showed great potential in reducing the number of copies of viral mRNA. Recently, it has been reported that essential oils demonstrated a similar antiviral effect on herpes viruses [41, 42].

Besides this, HMG showed the highest antiviral activity when added during the intracellular replication period. Our results are in accordance with that observed by Amoros et al. [43] during intracellular replication. Huleihel and Isanu (2002) [44] observed strong interaction between the propolis extracts and the surface of Vero cells but no direct interaction with herpes virus particles. The studies about the antiviral activity of propolis had revealed different modes of antiviral action [43, 44]. It remains to be determined whether the inhibitory effect of HMG is due to binding of some constituents of the propolis to viral proteins involved in host cell adsorption and penetration or is due to damage of the virions, possibly in their envelopes form, thereby impairing their ability to infect host cells.

On the other hand, the administration of HMG before the time of infection yielded the most significant inhibitory effect. Thus, the inhibitory action of EMG on viral replication could be through the IFN pathways. Recently, Melchjorsen et al. [45] showed that the initial response of the cells infected by HSV was the secretion of antiviral substances, such as defensins and nitric oxide, and, mainly, the production of cytokines, including IFNs and chemokines. The type IFNs (IFN-*αβ*) are key cytokines produced during the very first hours after HSV infection [45]. The outcome of HSV infections is dependent on the balance between virus propagation and effective immune response. Appropriate expression of IFNs, cytokines, and chemokines is essential for efficient host defense against infection [45]. Our study could suggest that HMG stimulated Vero cells in the production of interferon.

All the results obtained in our study are consistent with the images obtained by transmission electron microscopy (TEM), in which the presence of virions was not observed into the cytoplasm of cells inoculated with virucida sample. Besides this, no changes were observed in the nuclear membrane, which could suggest the formation of nucleocapsids [46] into the nucleus (Figure 2).

Propolis from different localities exhibited antiviral activity at different points in the viral replication [43, 44]. A synergism was demonstrated when binary flavones-flavonol combinations of extracts from propolis were tested against HSV. The results demonstrated that extracts from propolis were more active than its isolated constituents. The extracts containing many different components exhibited significant higher antiherpetic effects as well as higher selectivity indices than single isolate constituents [47].

For geopropolis from* Scaptotrigona postica* [5, 6], there are no studies about chemical composition. In our study, the main constituents found in HMG were flavones di-C-glycosides, showing vicenin-2 as the major constituent together with pyrrolizidine alkaloids as 7(3-methoxy-2-methylbutyryl)-9-echimidinylretronecine and caffeoylquinic acid-O-arabinoside. The geopropolis of* Melipona fasciculata* Smith from Baixada Maranhense, also in Maranhão state, Brazil, showed a different chemical composition, containing phenolic acids and hydrolyzable tannins (gallotannins and ellagitannins) as main constituents [4].

The antiviral activity of compounds identified in EMG, with the exception of echimidine derivatives, is known. The great potential of HMG in inhibition of HSV-1 indicated a significant effect on the stages of replication of the virus, what could be attributed to the high content of C-glycosylflavones. The C-glycosylflavonoids isolated from crude aqueous extract obtained from* Cecropia glaziovii* leaves exhibited antiherpes activity against human herpes virus types 1 and 2 by plaque reduction assay [48, 49]. Several flavone 6-C-monoglycosides showed potent,* in vitro* antiviral effect [50]. Apigenin, and not similar compounds like luteolin, naringenin, and quercetin, was active against enterovirus 71 infection [51], providing an evidence that one hydroxyl group difference in the B ring between apigenin and luteolin resulted in the distinct antiviral mechanisms [52]. Flavonoids showed highly effective antiviral activity against HSV-1 and HSV-2 [53].

Propolis extracts, which are rich in flavonoids and phenylcarboxylic acids, exhibited high levels of antiviral activity against HSV-2 in viral suspension tests [54]. Flavonoid and catechin derivatives showed inhibitory activity against herpes simplex virus type 1 and herpes simplex virus type 2 tested* in vitro* on RC-37 cells using a plaque reduction assay and exhibited high antiviral activity against both herpes viruses in viral suspension tests [55]. A Brazilian propolis that contained artepillin C as a main prenylated phenylpropanoid and chrysin as a main flavonoid showed not only direct anti-HSV-1 activity but also immunological activity against intradermal HSV-1 infection in mice [56]. Catechin-3-O-gallate [57, 58] and 3,4-dicaffeoylquinic acid exhibited significant antiviral action [59].

Thus, the great potential of HMG in reducing the copies of the viral DNA at low concentrations can be explained by the known antiviral activity of C-glycosylflavones, catechin-3-O-gallate, and 3,4-dicaffeoylquinic acid. The HMG acted inhibiting the viral replication as also inhibiting the entry of the virus into cells. This extract inhibited the replication of a virus of great importance to public health; however, more studies are necessary to elucidate the mechanisms of action and identify the molecules responsible for these effects.

## Figures and Tables

**Table 1:** Results obtained by RPHPLC-DAD-ESI-MS/MS analyses of geopropolis from *Scaptotrigona postica*: retention times (Rt), MS data for positive ion mode, and structure proposed.

	Rt	[M + Na]^+^	[M + H]^+^	Structure proposed
1	3.9	—	[M + H]^+^ 443 MS/MS—291	Catechin-3-O-gallate

2	5.6	[M + Na]^+^—365	[M + H]^+^—343	Caffeoyl glucoside

3	6.5		[M + H]^+^—415 MS/MS—397 (100), 379 (40), 361 (30), 331 (50)	7-Methoxy-5-hydroxy-8-C-flavone rhamnoside

4	15.5		[M + H]^+^ 430 MS/MS—412 (100), 385 (70), 367 (10), 315 (20)	7(3-Methoxy-2-methylbutyryl)-9-echimidinylretronecine or methoxy echimidine derivative

5	16.0		[M + H]^+^ 444 MS/MS—426 (100), 399 (20)	7(3-Ethoxy-2-methylbutyryl)-9-echimidinylretronecine or ethoxy echimidine derivative

6	16.5		[M + H]^+^ 619 MS/MS—601 (80), 583 (20), 565 (20), 535 (100)	Acacetin-di-C-acetyl dirhamnoside

7	17.9	[M + Na]^+^ 865	[M + H]^+^ 843 MS/MS—825 (100), 807 (40), 789 (20), 771 (30), 723 (60), 705 (50)	Apigenin-6,8-di-C-malonyl glucoside dihexoside isomer

8	19.2	[M + Na]^+^ 865	[M + H]^+^ 843 MS/MS—825 (80), 807 (40), 789 (30), 771 (20), 723 (70), 705 (100), 687 (40)	Apigenin-6,8-di-C-malonyl glucoside dihexoside isomer

9	20.2	[M + Na]^+^ 865	[M + H]^+^ 843 MS/MS—825 (100), 807 (50), 789 (20), 771 (30), 723 (60), 705 (70), 687 (40)	Apigenin-di-C-malonyl trihexoside isomer

10	20.4		[M + H]^+^ 857 MS/MS—839 (100), 803 (30), 737 (40), 719 (30)	Acacetin-di-C-malonyl trihexoside

11	21.1	[M + Na]^+^ 865	[M + H]^+^ 843 MS/MS—825 (90), 807 (50), 789 (30), 771 (25), 723 (100), 705 (60), 687 (10)	Apigenin-di-C-malonyl trihexoside isomer

12	22.6		[M + H]^+^ 563 MS/MS—417	Acacetin-8-C-arabinoside-7-O-rhamnoside

13	24.5	[M + Na]^+^ 865	[M + H]^+^ 843 MS/MS—825 (100), 807 (50), 789 (10), 771 (20), 723 (70), 705 (60)	apigenin-di-C-malonyl trihexoside isomer

14	27.0	[M + Na]^+^ 617 MS/MS—599 (100), 581 (20)	[M + H]^+^ 595 MS/MS—577 (100), 559 (30), 541 (20), 529 (40), 511 (50), 499 (30), 475 (30), 457 (60)	Vicenin-2

15	27.5	[M + Na]^+^—445 MS/MS—427 (100), 409 (18)	[M + H]^+^—423 MS/MS—405 (100), 387 (30), 369 (30), 357 (60), 327 (30)	Catechin arabinoside

16	28.0	[M + Na]^+^ 459 MS/MS—441	[M + H]^+^—437 MS/MS—419 (100), 401 (20), 383 (10), 371 (40), 341 (20)	Catechin rhamnoside

17	28.5		[M + H]^+^ 565 MS/MS—547 (100), 529 (70), 511 (50), 427 (60)	Apigenin-6,8-di-C-arabinoside glucoside

18	29.4		[M + H]^+^ 547 MS/MS—401	Chrysin-8-C-rhamnoside-7-O-rhamnoside

19	29.8		[M + H]^+^ 517 MS/MS—355 (80), 337 (100)	3,5-Dicaffeoyl quinic acid

20	31.0		[M + H]^+^ 595 MS/MS—577 (100), 567 (70), 551 (90)	Luteolin-8-C-caffeoyl rhamnoside

21	33.4		[M + H]^+^ 487	Caffeoylquinic acid-O-arabinoside
